# Toward an Inclusive, Congruent, and Precise Definition of Autoinflammatory Diseases

**DOI:** 10.3389/fimmu.2017.00497

**Published:** 2017-04-27

**Authors:** Per Wekell, Stefan Berg, Anna Karlsson, Anders Fasth

**Affiliations:** ^1^Department of Pediatrics, NU-Hospital Group, Uddevalla, Sweden; ^2^Department of Pediatrics, Institute of Clinical Sciences, University of Gothenburg, Gothenburg, Sweden; ^3^The Queen Silvia Children’s Hospital, Gothenburg, Sweden; ^4^Department of Rheumatology and Inflammation Research, Institute of Medicine, University of Gothenburg, Gothenburg, Sweden

**Keywords:** autoinflammatory diseases, definition and concepts, primary immunodeficiency, autoimmune diseases, rheumatology, pediatrics

## Abstract

Autoinflammatory disease was introduced as a concept in 1999, demarcating an entirely new group of diseases in clinical, immunological, and conceptual terms. During recent years, the preconditions for the definition of autoinflammatory conditions have changed. This includes the recent discovery of a number of monogenic autoinflammatory conditions with complex phenotypes that combine autoinflammation with defects of the adaptive and/or innate immune system, resulting in the occurrence of infection, autoimmunity, and/or uncontrolled hyperinflammation in addition to autoinflammation. Further, there are strong indications that classical IL-1-driven autoinflammatory diseases are associated with activation of adaptive immunity. As suggested by this development, we are of the opinion that an all-encompassing definition of autoinflammatory diseases should regard autoinflammatory conditions and innate dysregulation as inseparable and integral parts of the immune system as a whole. Hence, in this article, we try to advance the conceptual understanding of autoinflammatory disease by, proposing a modification of the definition by Daniel Kastner et al., which allows for a congruent and precise description of conditions that expand the immunological spectrum of autoinflammatory disease.

## Introduction

Autoinflammatory disease was introduced as a concept in 1999, demarcating an entirely new group of diseases in clinical, immunological, and conceptual terms (1). Over the years, the field has matured, and more specified phenotypes and deeper scientific knowledge of biological mechanism have successively changed the preconditions for an all-inclusive definition of autoinflammatory conditions. In particular, the characterization of monogenic autoinflammatory conditions with complex phenotypes that, for example, combine autoinflammation with autoimmunity, infection predisposition, or uncontrolled hyperinflammation calls for a broadened approach (2–4). In retrospect, a clue to the discovery of complex phenotypes already existed in the classic periodic fever syndrome mevalonate kinase deficiency (MKD), or hyper-IgD (HIDS) syndrome, that often combines an autoinflammatory phenotype with recurrent bacterial infections, autoimmunity (autoimmune cytopenia), and marked systemic inflammation (5). Here, we map the recent developments in the field and discuss the conceptual challenges that these developments entail, with the goal to propose an updated definition of autoinflammatory diseases that is inclusive (broad) (6) rather than exclusive (narrow) (7).

Autoinflammatory diseases were initially defined by Michael McDermott and Daniel Kastner in 1999 as “conditions characterized by seemingly unprovoked episodes of inflammation, without high-titer of autoantibodies or antigen-specific T-cells” (1, 8). This formulation made a clear division between autoinflammation and autoimmunity, at that time an important distinction to make.

Seven years later, McGonagle and McDermott proposed that immunological diseases ought to be conceived as a continuum with “pure monogenic autoinflammatory diseases” at one end and “pure monogenic autoimmune diseases” at the other (9). This continuum model moved the understanding of immunological diseases forward by entwining the concept of autoinflammation with that of autoimmunity and applying the concept of autoinflammation not only to monogenic diseases but also to polygenic diseases. It also recognized that polygenic and multifactorial diseases might have both an autoinflammatory and an autoimmune component (9).

In 2010, Daniel Kastner et al. proposed that autoinflammatory diseases are “*clinical disorders marked by abnormally increased inflammation, mediated predominantly by cells and molecules of the innate immune system, with a significant host predisposition*” (10). This definition recognizes that cells and molecules of innate immunity, including, monocytes, neutrophils, and NOD-like receptors are fundamental in autoinflammatory disease (10). This is the definition that is generally accepted and used at present.

## The Expanding Disease Spectrum of Autoinflammatory Diseases

Today, several studies show that classical IL-1-driven autoinflammatory diseases are associated with activation of adaptive immunity (11–13). Further, a number of recently defined monogenic autoinflammatory conditions have complex phenotypes that combine autoinflammation with defects of the adaptive and/or innate immune system, resulting in the occurrence of infection, autoimmunity and/or uncontrolled hyperinflammation in addition to autoinflammation (Table 1) (14–19).

**Table 1:** **Characterization of monogenic autoinflammatory diseases with complex phenotypes**.

Secondary component	Susceptibility to infections	Autoimmunity	Uncontrolled hyperinflammation as in HLH
Autoinflammation			
APLAID	APLAID (15)		
HOIL-1	HOIL-1 (14, 22)		
SIFD	SIFD (16, 24)		
AGS		AGS (17, 26–36)	
NLRC4-MAS			NLRC4-MAS (18)
MKD	MKD (5)	MKD (5)	MKD (5)

*HLH, hemophagocytic lymphohistiocytosis; APLAID, autoinflammation and PLCγ2-associated antibody deficiency and immune dysregulation; HOIL-1, heme-oxidized IRP2 ubiquitin ligase 1 deficiency; SIFD, sideroblastic anemia with B-cell immunodeficiency, periodic fever, and developmental delay; AGS, Aicardi–Goutières syndrome; NLRC4-MAS, NLR family CARD domain containing 4-macrophage-activating syndrome; MKD, mevalonate kinase deficiency*.

The IL-1-driven diseases activate adaptive immunity by differentiation of CD4+ T cells toward a Th17 response. For example, in patients with cryopyrin-associated periodic syndromes (CAPS), there is an increased number of Th17 cells and IL-17 in the blood as well as an increased percentage of Th17 cells in skin biopsies (12, 13). Therapeutic IL-1-blockade not only leads to inhibition of the IL-1-mediated inflammatory symptoms but also to decreased numbers of Th17 cells, indicating that (auto)inflammatory dysregulation activates the adaptive immune system and that this activation is part of the autoinflammatory disease phenotype. In addition NLRP3 inflammasome assembly in human CD4+ T cells promotes interferon-γ (IFN-γ) production and T helper cell (Th1) differentiation. NLRP3 assembly requires intracellular C5 activation and stimulation of C5a receptor 1 (C5aR1) demonstrating that NLRP3 inflammasome activity is an integral component of normal adaptive Th1 responses (20). Further, Th1 cells in patients with CAPS take part in increased production of IL-1β and IFN-γ, which suggests an essential contribution of the adaptive immune system to the immunological phenotype in patients with CAPS (20). This direct coupling between the innate and adaptive system in IL-1-driven diseases, as in MKD, challenges the concept that there are “pure autoinflammatory diseases” as conceived in the continuum model (9).

Diseases defined by mutations in the innate immune system that leads to phenotypes in which autoinflammation is combined with susceptibility to infections have recently been described (14–16). One such disease is autoinflammation and PLCγ2-associated antibody deficiency and immune dysregulation (APLAID), caused by missense mutations in (the gene) *PLCG2*. This condition has clinical manifestations of inflammation (recurrent skin blistering, pneumonitis, and ocular inflammation) in combination with susceptibility to infections (recurrent sino-pulmonary infections) due to low concentrations of IgA and IgM (15). In contrast, distinct in-frame deletions in *PLCG2* are associated with another syndrome, the PLCγ2-associated antibody deficiency and immune dysregulation syndrome (PLAID) (21). A second condition in this group is heme-oxidized IRP2 ubiquitin ligase 1 deficiency (HOIL-1 deficiency). This disease has a phenotype that combines autoinflammatory symptoms (recurrent episodes of fever and systemic inflammation) with reduced numbers of memory B cells, resulting in an impaired response to pneumococcal polysaccharides resulting in recurrent infections (14, 22). Interestingly, the immunological consequence of the mutation is different in different cells: on the one hand, HOIL-1-deficient lymphocytes and fibroblasts show compromised activation of NF-κB signaling in response to IL-1β, in keeping with the described immunodeficiency. On the other hand, HOIL-1-deficient monocytes display enhanced sensitivity to IL-1β and produce large amounts of IL-6 and MIP-1α, which can explain the autoinflammatory manifestations (23). A third example is a disease caused by a mutation in the *TRNT1* gene, which shows congenital sideroblastic anemia with B-cell immunodeficiency in combination with periodic fever and developmental delay (16, 24). These examples make clear that autoinflammatory diseases also may encompass a dimension of deficiency in the adaptive immune system that results in infection proneness.

Type I interferonopathies display both autoinflammatory and autoimmune components. Rare monogenic type I (INF-α and INF-β) interferonopathies comprise a group of diseases with heterogeneous phenotypes. They share a pathological mechanism in which mutations lead to chronic type I interferon secretion, resulting in autoinflammatory symptoms with a “spill-over into autoimmunity in some cases” (25). There are a few case studies that demonstrate a true pathogenic role of the autoantibodies observed [e.g., neuromyelitis optica, and SLE in Aicardi–Goutières syndrome (AGS)] (26–31) and several examples of a characteristic spectrum of autoantibodies (17, 32, 33) with an unclear pathogenic role. Furthermore, there is an overrepresentation of autoimmune diseases in a large cohort of AGS (34). The strongest support for autoimmune disease in interferonopathies is cytopenias and early-onset SLE in patients with spondyloenchondrodysplasia due to mutations in *ACP5* (35, 36). This suggests that autoimmune components are not restricted to polygenic diseases in the autoinflammatory—autoimmune disease spectrum, as proposed by McGonagle and McDermott, but may be a facet also of monogenic autoinflammatory disease.

Uncontrolled hyperinflammation as in hemophagocytic lymphohistiocytosis (HLH) has been described in a few patients with classical monogenic autoinflammatory diseases but without significant mortality (37), including TNF-receptor-associated periodic syndrome (38), familial Mediterranean fever (39), MKD (5), and CAPS (40, 41) and is also recognized as a quite common complication of systemic juvenile idiopathic arthritis flares (42). Mutations in *NLRC4* may lead to a phenotype with familial cold autoinflammatory syndrome as seen in CAPS, but also to a phenotype that combines early-onset autoinflammatory periodic fever syndrome with life-threatening uncontrolled hyperinflammation as in HLH during the most severe febrile episodes (18). Intrinsic macrophage activation is likely to drive the HLH by uncontrolled secretion of IL-18 in NLRC4-associated disease (p.V337S and p.V341A). This disease mechanism is contrasted by familial hemophagocytic lymphocytosis 1–5, in which the uncontrolled inflammation is a consequence of cytolytic failure of CD8 T lymphocytes and natural killer cells. Hence, the risk of uncontrolled hyperinflammation may be part of autoinflammatory diseases and needs to be echoed in our understanding of autoinflammatory diseases and in an updated definition of those disorders.

Conditions of the immune system with complex phenotypes that combine several immunological dimensions are not unique to autoinflammatory diseases. For example, the monogenic immunodeficiency chronic granulomatous disease (CGD) caused by mutation in genes that code for the phagocyte NADPH oxidase and resulting in aberrant production of reactive oxygen species (ROS) is associated not only with increased susceptibility to infection but also with sterile inflammation in organs like the gastrointestinal tract, urogenital tract, and lungs (43–45). Normal levels of NADPH oxidase-derived ROS are likely to control inflammation (46), while dysregulation, causing for example, colitis, is responsive to IL-1 blockade (47, 48). Hence, CGD presents with a phenotype comprising increased susceptibility to infection due to deficiency in the innate immune system, in turn associated with autoinflammatory traits. However, such conditions, dominated by predisposition to infection, albeit with an autoinflammatory component, are not addressed in the previously proposed definition of autoinflammatory diseases.

## Toward a Comprehensive Definition of Autoinflammatory Conditions

The definition of autoinflammatory diseases proposed by Daniel Kastner in 2010 is formulated in an open way that is compatible with the recent discoveries of complex autoinflammatory phenotypes and concurrent activation of adaptive immunity in classical monogenic autoinflammatory diseases. Nevertheless, the Kastner definition does not specifically address the complex phenotypes that were not known at the time. Today, an all-encompassing definition of autoinflammatory diseases should regard autoinflammatory conditions and innate dysregulation as inseparable and integral parts of the immune system as a whole. Only such a definition allows for a congruent and precise description of conditions that expand the immunological spectrum of autoinflammatory disease. Even though a new definition is called for, it is a difficult task to formulate a definition that can be generally agreed upon among experts in the field. With the aim to be inclusive in addition to be congruent and precise, we propose the following modification of the definition by Daniel Kastner:

Autoinflammatory diseases are immunological diseases defined by abnormally increased inflammation, driven by dysregulation of molecules and cells of the innate immune system with a host predisposition as necessary and sufficient criteria, frequently associated with activation of the adaptive immune system and potentially with immune dysfunctions such as susceptibility to infections, autoimmunity or uncontrolled hyperinflammation.

## Author Contributions

PW wrote the first draft of the manuscript. All authors made substantial contribution of the conception of the work, the drafting the work, and revising it critically for important intellectual content; have approved the submitted version of the manuscript; and have agreed to be accountable for all aspects of the work in ensuring that questions related to the accuracy or integrity of any part of the work are appropriately investigated and resolved.

## Conflict of Interest Statement

The authors declare that the research was conducted in the absence of any commercial or financial relationships that could be construed as a potential conflict of interest.
